# Indicators and methodologies for assessing urban agglomeration sustainability

**DOI:** 10.1016/j.isci.2025.112927

**Published:** 2025-06-18

**Authors:** Yinghui Shao, Wei Han, Min Jin, Lizhe Wang

**Affiliations:** 1 School of Computer Science, China University of Geosciences, Wuhan, China; 2 Department of Electronic Information Engineering, Wuhan Business University, Wuhan, China

**Keywords:** Earth sciences, Environmental science

## Abstract

Urban agglomerations are crucial for achieving the United Nations Sustainable Development Goals (SDGs). However, uncertainties remain in the indicators and methodologies used to measure their sustainability. This paper establishes and analyzes the SDGs network at national and urban agglomeration levels, highlighting that urban agglomerations are key scale to achieving sustainable development but are far more complex than a single city scale. Then, this paper proposes an assessment framework for urban agglomeration, which includes indicators, methodological approaches, and validation processes. By introducing the multi-level coupling coordination degree, various influencing mechanisms are integrated into the evaluation stage. In addition, this paper constructs redundancy and sensitivity analyses and establishes a correlation network between indicators and SDGs to collectively validate the robustness and international compatibility of the indicator system. This study provides strong support for understanding and promoting the sustainable development of urban agglomerations, contributing to the achievement of the 2030 Agenda.

## Highlights

• Uncovers complex relationships in urban agglomeration sustainable development
• Develops a framework integrating indicator methods and validation for assessment
• Applies indicators and methods to middle Yangtze River urban agglomeration
• Proposed indicators capture core challenges faced by urban agglomerations

## Graphical abstract

**Figure:**
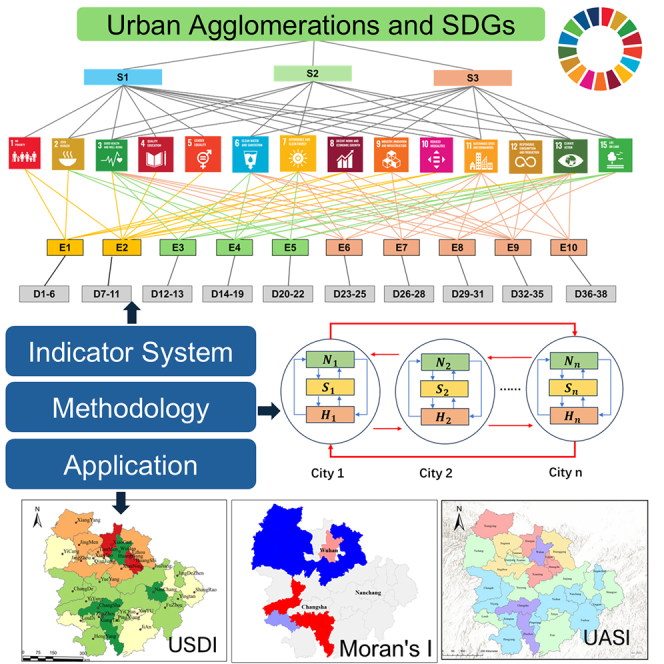

## Introduction

Extensive industrial activity, resource extraction, and urban sprawl have placed immense pressure on ecosystems, exacerbating sustainable development challenges worldwide, including geological disaster, mining pollution,^1^ soil erosion,^2^ and environmental degradation.^3^ The United Nations adopted 17 ambitious Sustainable Development Goals (SDGs) aimed at promoting sustainable economic, social, and environmental development globally.^4^ The SDGs translated the abstract concept of sustainable development into concrete and measurable standards, which lays a solid foundation for the quantification of sustainable development.^5^

As the largest concentration of human socio-economic activities, cities have been recognized as one of the basic units for assessing the level of sustainable development.^6^^,^^7^ The SDG 11 (Sustainable Cities and Communities) offers specific indicators for assessing urban sustainability to a certain degree.^8^ However, there are complex nonlinear relationships among SDGs, which are manifested as trade-offs and synergies between the goals.^9^^,^^10^ Especially at the nexus of climate change (SDG 13) and economic development (SDG 8), climate change intensifies income risk heterogeneity within and across countries by impacting natural resources, agricultural production, and infrastructure, resulting in both direct and indirect shocks to economic activity.^11^ Therefore, it is difficult to capture the interconnections and dynamics inherent in regional sustainable development from a single city perspective.^12^

Urban agglomeration refers to a spatial structure emerging at the mature stage of urban development, including the Great Lakes Urban Agglomeration, the British Urban Agglomeration, and the Beijing-Tianjin-Hebei Urban Agglomeration.^13^ These regions play a critical role in promoting regional economic integration, enhancing national or regional competitiveness, and addressing global challenges such as climate change and resource scarcity.^11^^,^^14^ In this context, population migration and the accelerated transmission of information over long distances have heightened interactions among various elements. For example, hidden pressures on cultivated land are redistributed through interregional trade flows.^15^ However, these interactions also add complexity to achieving the SDGs, as cities within agglomerations are embedded in interconnected urban networks rather than functioning independently. To address this gap, it is essential to consider the interdependence among cities, such as shared environmental burdens, policy spillovers, and competition for resources.

In sustainable development assessment, many studies have explored the application of different methods. The existing literature can be broadly categorized into top-down methods and bottom-up methods. Top-down approaches are usually based on global standards or policy frameworks, such as the SDGs, which provide unified evaluation criteria and indicator systems for countries and regions. For example, one study identified nearly 50 specific indicators aligned with the 17 SDGs and analyzed spatial performance in social, economic, and environmental scores.^12^ Another study conducted in Hainan Province localized 73 indicators across 13 SDGs for 18 cities, focusing on the SDG composite index and its associated spatial spillover effects.^16^ However, this approach often overlooks the specific characteristics and needs of diverse cities, and may neglect indicators outside of the SDGs. Additionally, disparities in data access, quality, and availability exist across different regions and countries.

On the other hand, the bottom-up approach develops indicators by emphasizing the specific development needs and characteristics of urban areas. For example, A case study of 35 cities in China constructed an indicator system to evaluate urban ecosystem resilience in the DPSIR framework.^17^ Another example comes from the Wuhan Metropolitan Area, where researchers quantitatively assessed the integration of soil, water, and air environments to establish an Integrated Perception Ecological Environment Indicator.^18^ Other studies use the Ha-Chang and Mid-Southern Liaoning urban agglomerations as examples to study the time series evaluation of sustainable development, introducing the growth rate and annual growth trend of indicators.^19^ However, differences in researchers’ perspectives and their interpretations of the urban context can render the selection and development of indicators more subjective and variable. This diversity can complicate direct comparisons among various sustainability assessments.

To address these gaps, this paper uncovers the complex interrelations underlying the sustainable development of urban agglomerations from the network perspective first, which manifest not only in the spatial clustering effects of these regions but also in the complex interactions among various SDGs. Second, this paper establishes the “Indicator-Methodological Approaches-Validation Processes” framework for assessing the sustainable development of urban agglomerations, addressing the lack of unified standards and synergy mechanisms across various thematic assessment operations due to data availability and inconsistent sustainable development evaluation objectives under different socio-economic and cultural conditions. Finally, the indicators and methodologies proposed in this study were applied to the Yangtze River Middle Urban Agglomeration (YRMUA), facilitating an in-depth analysis of its sustainable development status and validating the effectiveness of the proposed indicator system and methodologies.

## Results

### The role of urban agglomerations in China’s sustainable development

**Figure 1:**
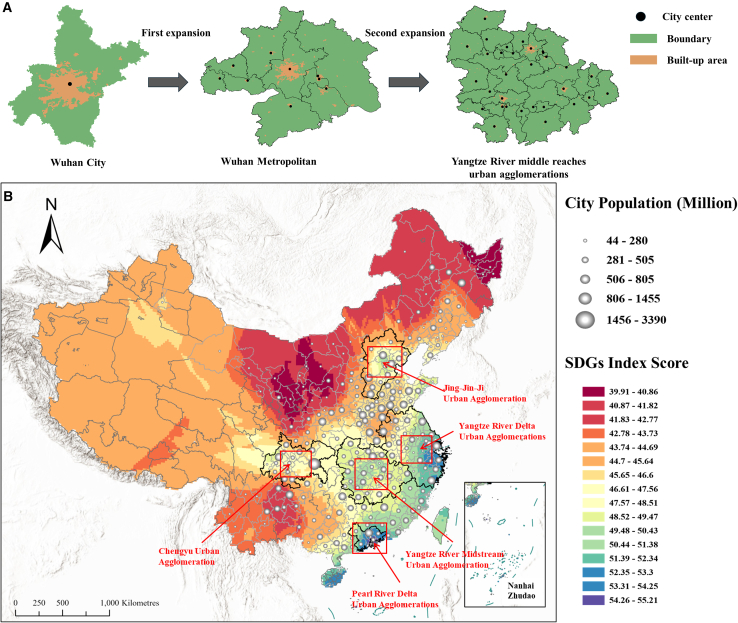
Sustainable development and spatial patterns of urban agglomerations in China (A) Urban agglomerations in the middle reaches of the Yangtze River: these areas gradually develop from a single city into a metropolitan region, ultimately forming an interconnected and coordinated urban agglomeration. (B) Spatial heterogeneity of China’s SDGs progress: the five major urban agglomerations in China significantly overlap with regions exhibiting higher SDG scores, indicating that the sustainable development of urban agglomerations plays a crucial role in enhancing the country’s overall sustainability.

The spatial pattern of sustainable development in China is characterized by a high east and a low west and a high south and a low north. According to the SDG assessment scores of more than 200 cities in China, the five major urban agglomerations attain higher SDG scores, whereas the levels in the surrounding areas exhibit a gradual decline with increasing distance from the central urban area (Figure 1B). This trend shows that the sustainable development of urban agglomerations is a driving force behind the country’s SDGs and holds a critical position. The urban agglomeration in the middle reaches of the Yangtze River is in the center of China (Figure 1A), connecting the eastern coast and the western inland, which is one of the critical regions for achieving sustainable regional development in China.

**Figure 2:**
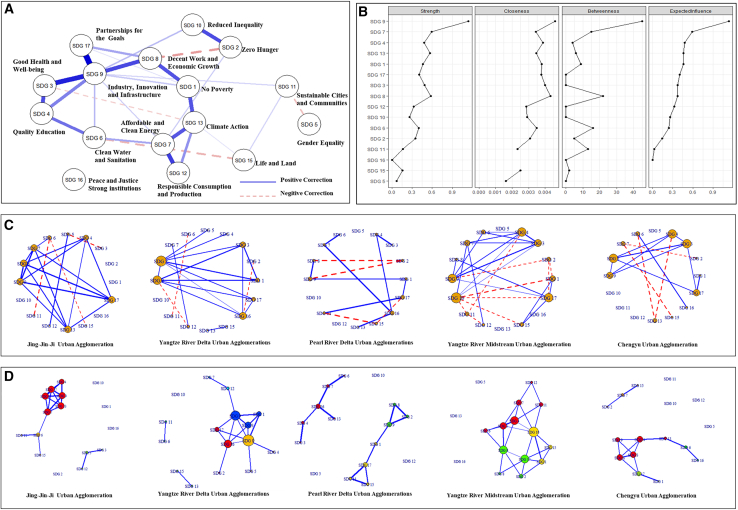
SDGs network analysis and community detection (A) The SDG network in China is primarily characterized by synergies, with a relatively low proportion of trade-offs. (B) Centrality measurements of nodes within the interrelationship network of the SDGs indicate that SDG 9 is the most influential node. (C) The interactive network of SDGs in the five major urban agglomerations in China reveals that the trade-off effect is most significant in the urban agglomeration in the middle reaches of the Yangtze River, highlighting the complex balancing challenges faced in this region. (D) Community detection of the interactive SDG network in the five major urban agglomerations in China demonstrates the relationships among the SDGs.

Network-based approaches have been used in previous studies to analyze the intricate relationships,^20^ where positive linkages indicate synergies and negative linkages indicate trade-offs. By constructing the SDGs network on the national scale, this study reveals a significantly higher proportion of positive connections compared to negative connections (Figure 2A). This finding indicates a favorable trend in China’s advancement of the SDGs, underscoring robust synergies across multiple goals. There is a positive correlation between SDG 9 (Industry, Innovation and Infrastructure) and each of the following: SDG 3 (Good Health and Well-being), SDG 17 (Partnerships for the Goals), and SDG 4 (Quality Education). Conversely, significant negative correlations exist between SDG 6 (Clean Water and Sanitation) and SDG 15 (Life and Land), between SDG 2 (Zero Hunger) and SDG 8 (Decent Work and Economic Growth), between SDG 5 (Gender Equality) and SDG 11 (Sustainable Cities and Communities) and between SDG 3 (Good Health and Well-being) and SDG 13 (Climate Action), indicating potential competition or conflict regarding water resource management, ecosystem protection, and industrial growth. SDG 9 (Industry, Innovation, and Infrastructure) exhibits the highest centrality measures (strength, closeness, betweenness, and expected influence) in the network (Figure 2B). This indicates that SDG 9 plays a crucial role in the comprehensive realization of the SDGs.

Although positive connections among the SDGs are more prevalent than negative connections at the national scale, the synergies and trade-offs among the SDGs at the urban agglomeration scale are more pronounced than at the national scale (Figure 2C). Notably, the SDGs network structure of the Pearl River Delta urban agglomeration is distinctly defined, whereas the associative mechanisms in the Yangtze River Midstream urban agglomeration are notably complex, featuring multiple potential connections among diverse goals. This indicates that the conflicts and synergies among the various goals are more pronounced in this region (Figure 2D).

### Summary of indicators and methodologies

**Figure 3:**
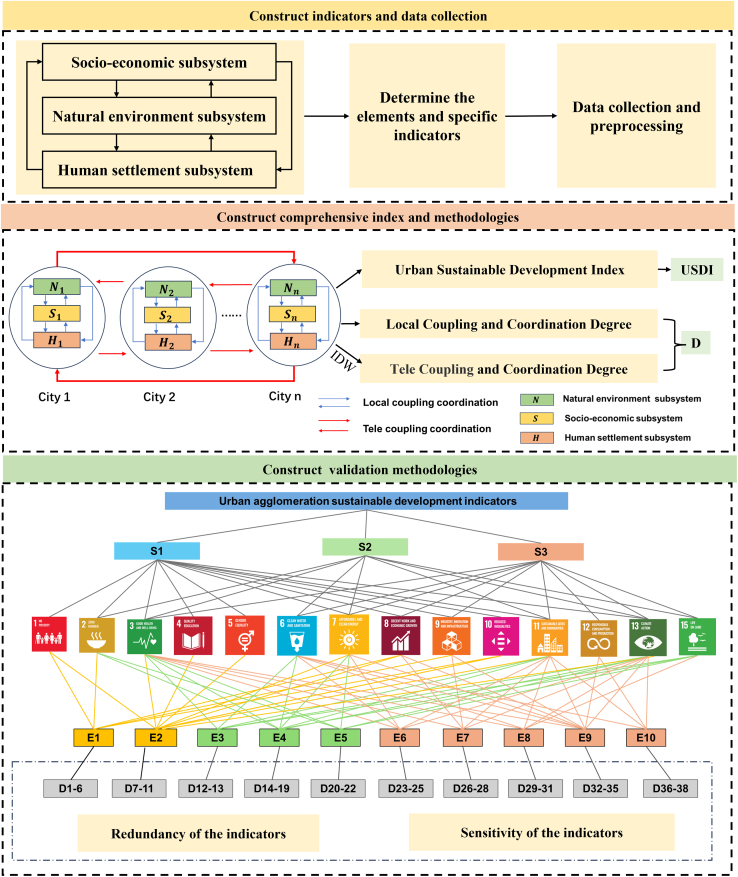
Urban agglomeration sustainable development indicators and methodologies The procedure is divided into three parts: constructing indicators and data collection; designing multi-level coupling coordination degree based on the Coupling Coordination Degree for assessing sustainable development; and developing validation methodologies, including indicator redundancy, sensitivity validation and establishment of SDGs-indicator linkage networks.

To reveal the complexity of sustainable development in urban agglomerations, this paper analyzes the pattern of sustainable development in China and constructs the SDGs network at both national and urban agglomeration scales in the previous subsection. Then, this paper proposes a framework for assessing sustainable development in urban agglomerations (Figure 3). The framework encompasses three key components: indicator,^21^ methodological approaches,^22^ and validation processes.^23^ In the indicator establishment stage, this paper introduces a “subsystem-element-indicator” system. Building upon the social, economic, and environmental dimensions and considering the characteristics of high population density and intensive economic activities, this paper categorizes urban agglomerations into three subsystems: the Natural Environment Subsystem (NES), the Socio-Economic Subsystem (SES), and the Human Settlement Subsystem (HSS). Within these subsystems, this paper identifies 10 elements and 38 specific indicators.

In the methodological approaches section, this paper presents the multi-level coupling coordination degree to elucidate the interactions between multiple subsystems within urban agglomeration. This paper establishes the Urban Agglomeration Sustainability Index (UASI). The UASI is quantitatively assessed through three key dimensions: sustainability performance across three subsystems, the Urban Sustainable Development Index (USDI), and the multi-level coupling and coordination degree at the urban agglomeration scale (details in the STAR Methods section).

This paper designs a corresponding validation process—except for the effectiveness and robustness of the assessment framework, the quantitative analysis approach “subsystem-element-indicator-sustainable development goal” network is proposed, which establishes an effective link with international sustainable development standards. In addition to SDG 14, we excluded SDG 16 and SDG 17 during the validation phase. SDG 17 primarily pertains to macro-level global governance and international cooperation, which fall outside the scope of our study on the internal development of urban agglomerations. Similarly, SDG 16 was excluded due to its lack of significant positive or negative correlations with other SDGs (Figure 2A). The “Indicator-Methodological Approaches-Validation Processes” framework for the sustainable development of urban agglomerations proposed is applied in the YRMUA finally.

Notably, due to differences in the definitions of cities and urban agglomerations between China and other countries, in this paper “city” refers to the administrative area that includes both the built-up area and the surrounding rural regions. The “urban agglomeration” refers to a group of cities spanning multiple administrative regions, which emphasize coordinated regional development. This cross-regional characteristic makes it impossible to regard the level of sustainable development of an urban agglomeration as simply the sum of the development levels of several cities, and the coordination and balance between different regions must be considered in the assessment. In addition, it brings a series of problems such as missing data and mismatched indicators, which increase the difficulty of the assessment.

### Case study of indicators and methodologies in the YRMUA

This section progressively describes the intermediate steps in constructing the UASI, which is a step-by-step process of combining them from the bottom up. First, the score of the three subsystems is calculated based on Equation 4 in this paper. The USDI for individual cities is obtained using Equation 5. Subsequently, the coupling mechanisms within and between cities were quantified according to Equations 6, 7, 8, 9, 10, 11, 12, and 13, based on the methodology of multi-level coupling co-ordination degrees. Ultimately, the UASI was established based on Equation 14, which describes the sustainability model of the urban agglomeration.

**Figure 4:**
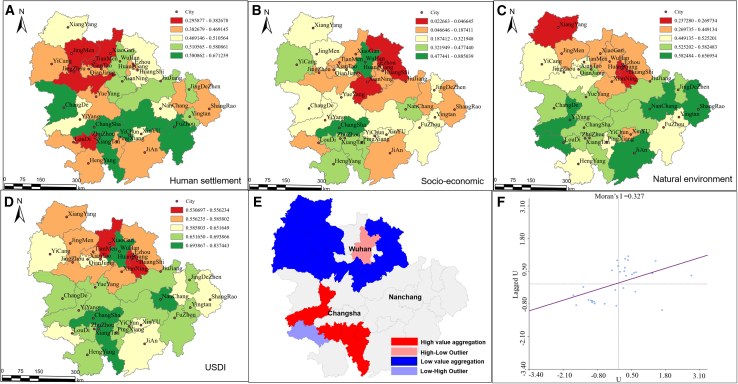
Sustainable development analysis of the Yangtze River Midstream Urban Agglomeration (A–C) The scores of the three subsystems of urban agglomeration in the YRMUA. (D) Sustainable development scores of cities in the YRMUA. (E and F) Spatial autocorrelation of sustainable development by Moran’s I in the YRMUA.

In the performance of subsystems within the YRMUA, a negative trend is observed between the SES and the NES, as well as between the HSS and the NES (Figures 4A–4C). This finding suggests that improvements in SES and HSS conditions may place additional pressure on natural resources and environmental quality. In contrast, the relationship between the HSS and SES is primarily characterized by a positive correlation, indicating that advancements in socio-economic conditions are generally associated with enhancements in human living environments. The USDI is a comprehensive index for evaluating the sustainable development of a single city, derived based on the calculation of three interconnected subsystems. Hubei Province predominantly exhibits a “low-low” clustering pattern, while Hunan Province is characterized primarily by “high-high” clustering. Furthermore, the quantity of “low-low” clustered cities significantly exceeds that of “high-high” clustered cities, indicating that regions with lower levels of sustainable development tend to form spatial agglomerations more readily (Figures 4D–4F).

**Figure 5:**
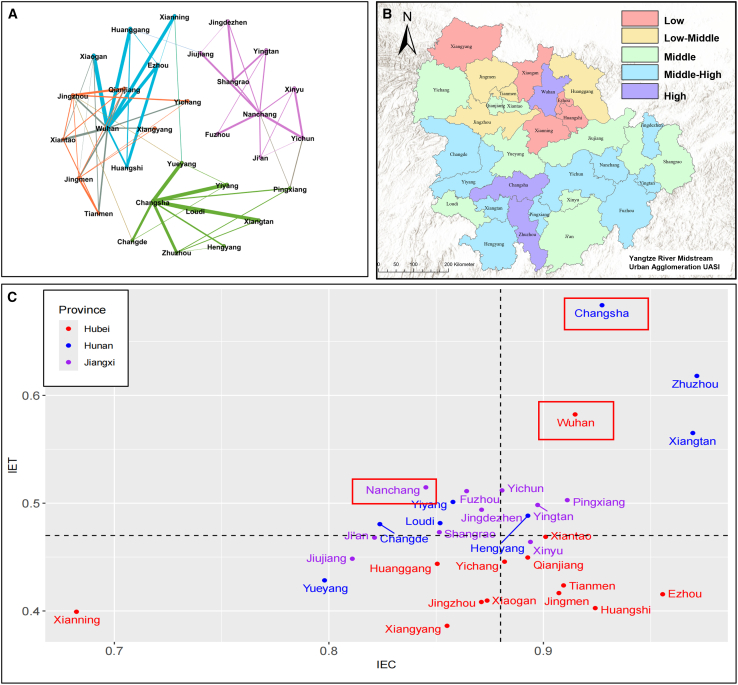
Patterns among urban agglomerations (A) Human mobility networks and community detection, where colors denote divisions into four distinct communities. (B) The Urban Agglomerations Sustainability Index for the YRMUA. (C) The four quadrants of the multi-level coupling coordination degree among and within cities.

Based on natural environment, socio-economic, human settlement, and USDI performance, the multi-level coupling coordination degree is used to measure various influencing mechanisms (as defined in Equations 8 and 12). The 31 cities within the YRMUA are divided into four categories according to the rules (Figure 5C).

In the first quadrant’s coupled development model, the coupling level exceeds 0.88, and the harmonization level exceeds 0.47 in ideal development scenarios, with half of these cities located in the Hunan Province. These cities demonstrate outstanding performance in coupling and development, positioning them favorably for a sustainable trajectory. These areas represent the most developed regions within the YRMUA, where socio-economic development is decoupled from resource consumption and environmental degradation. Consequently, these cities should provide technical and financial support to their surrounding regions. In the second quadrant’s uncoupled development model, the coupling level is below 0.88, while the cooperation level exceeds 0.47, primarily in Hunan and Jiangxi. Most of these cities possess excellent natural environments but exhibit relatively weak human settlements or socio-economic subsystems. This indicates an imbalance between the natural environment and socio-economic conditions in these areas. Sufficient funding is essential to support infrastructure development, enhance habitat quality, and promote socio-economic growth.

In the third quadrant’s uncoupled and underdeveloped model, the coupling level is below 0.88, and the harmonization level is below 0.47, with most cities located in Hubei Province. Cities in this model exhibit low socio-economic levels and limited capacity for sustainable development. These cities must strengthen their connections with developed regions to obtain technical support and foster economic development. In the fourth quadrant’s coupled and underdeveloped model, cities demonstrate balanced development across the three subsystems but generally exhibit weaker performance. Therefore, it is crucial for local governments to tailor their investments to support development based on specific needs and circumstances.

The UASI is derived from the integration of the USDI and the “multi-level coupling coordination degree” to assess urban agglomeration performance. This study categorizes cities into five levels based on the UASI (Figure 5B). In Hubei Province, a notable siphoning phenomenon exists, where Wuhan serves as the dominant city, yet neighboring cities exhibit low scores. The UASI in Jiangxi Province reflects homogeneity in sustainable development, with no significant high or low scores observed. In Hunan Province, Changsha acts as a significant driving force, influencing its neighboring cities.

This paper explores the patterns of material and energy flow in urban agglomerations by utilizing population migration data to construct an urban agglomeration network and detect community structures.^24^ Population migration within the YRMUA was uneven, resulting in the formation of distinct communities characterized by relatively weak interconnections (Figure 5A). Wuhan and its surrounding cities have formed several communities characterized by weak connections. In contrast, Changsha and its surrounding cities form a large community, with Changsha serving as the core node. Conversely, the communities formed in Nanchang and its surrounding cities lack distinct core nodes and exhibit relatively uniform characteristics.

In the case of the Wuhan Metropolitan Area, emphasizing the promotion of integration among cities could enhance resource efficiency and foster collaboration, considering the weaker interconnections observed between communities. Similarly, in the Poyang Lake Metropolitan Area, nurturing core cities like Nanchang could stimulate overall development. By strengthening Nanchang’s role as a central node and promoting collaboration among surrounding cities, the entire urban agglomeration could leverage its collective strengths, achieving more balanced and resilient growth.

### Validation of sustainable development indicators and methodologies

While the case study of the YRMUA has provided valuable insights, it is essential to validate these indicators and methodologies to confirm their robustness and reliability. The indicators and methodologies are validated through redundancy and sensitivity analyses. Redundancy analysis assesses the consistency of subsystems, while sensitivity analysis examines the stability and reliability of the indicator system. The indicator system is considered valid if redundancy ≤0.5 and sensitivity ≤5.^25^ At the subsystem level, the average redundancy values for the socio-economic system, the natural environment, and human habitation are 0.21, 0.38, and 0.44, and the whole indicator system redundancy is 0.42. This indicates that the three subsystems demonstrate acceptable redundancy levels. Furthermore, cross-system correlation analysis at the element level reveals a significant positive correlation between social and traffic elements, while geographical and open-space elements, as well as industrial-pressure and open-space elements, exhibit significant negative correlations (Figure S1). These findings reinforce the notion that complex multi-scale interactions exist among various sustainability dimensions, highlighting the importance of a nuanced approach when assessing sustainability at urban agglomeration scale.

The sum sensitivities of three subsystems are 0.58, 0.7, and 0.9, and the whole indicator system sensitivity is 0.76. In the aspect of indicator, our findings indicate that slope, gas supply penetration, and water supply permeability had the highest contributions at the element level, whereas gas supply penetration rate and water supply permeability exhibited the highest contributions to the overall indicator system (Table S4). Notably, the identified key sensitive indicators do not necessarily require upgrades, they should be further analyzed in conjunction with the development objectives of urban agglomerations. To enhance the sensitivity of the analysis while preserving the original data distribution, we set the small adjustment to 0.5 in the overall index system sensitivity analysis. This amplification of variations allowed for a more precise assessment of the impact of small perturbations.

By integrating redundancy contribution and sensitivity analysis, we classified the indicators into four distinct categories. (1) “Low Redundancy–Low Sensitivity Type” exhibits the limited impact on urban sustainability assessment. These indicators primarily serve as background descriptors rather than key evaluative factors, including the annual growth rate of per capita GDP, urban registered unemployment rate, and per capita education expenditure in our indicator system. (2) “Low Redundancy – High Sensitivity Type” are critical drivers of urban sustainability. Their removal may lead to significant loss of information, as they provide unique insights into the system, including urban-rural income disparity, slope, and distance to water resources in our indicator system. (3) “High Redundancy – High Sensitivity Type” may contain overlapping information; they still serve as valuable references and can function as auxiliary decision-making variables, including temperature variation rate, gas supply coverage, and water supply coverage. (4) “High Redundancy – Low Sensitivity Type” is the most suitable indicator for elimination or integration, including the proportion of the tertiary industry, per capita disposable income of urban residents, and per capita disposable income of rural residents in our indicator system (detail in Table S5).

**Figure 6:**
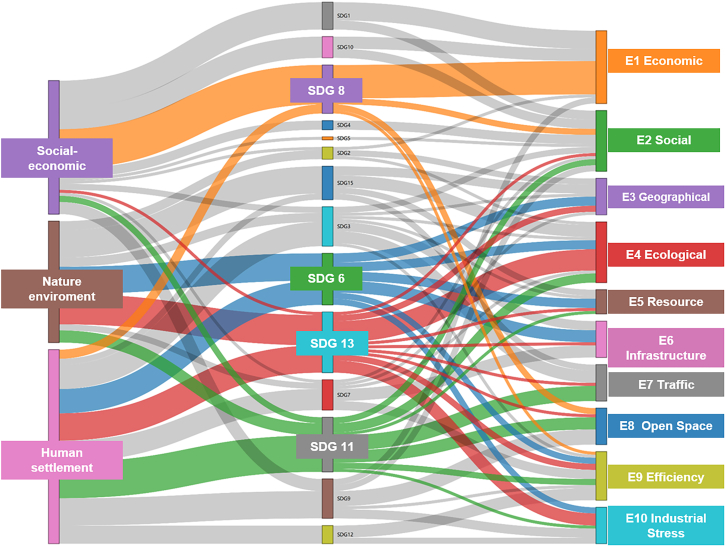
Correlation network between indicator systems and SDGs

This paper proposes a correlation network between indicators and SDGs. The proposed network is based on a comprehensive literature review and theoretical hypotheses (Figure 6; Table S2). These indicators specifically address SDG 3 (Health and Well-being), SDG 6 (Clean Water and Sanitation), SDG 8 (Economic Growth and Employment), SDG 11 (Sustainable Cities and Communities), and SDG 13 (Combating Climate Change). These indicators closely align with the urban development needs outlined in the World Cities Report 2022, demonstrating their relevance to the sustainability concerns of urban agglomerations.

## Discussion

Given the complexity of urban systems and their extensive linkages with the external environment, effectively addressing these issues at the individual city scale is challenging.^26^ Urban agglomerations represent a novel meso-scale unit of study capable of addressing these complex challenges more comprehensively.^4^^,^^13^ This study contributes to the growing body of research on sustainable development in urban agglomerations by addressing several notable gaps.

First, although previous studies have acknowledged the importance of urban agglomerations, they have primarily relied on qualitative analyses and offer limited empirical evidence. This study addresses this gap by constructing SDGs interaction networks at both national and urban agglomeration scales, revealing the spatial heterogeneity and multi-dimensional interlinkages of sustainable development performance across regions from both qualitative and quantitative perspectives.

Second, many existing evaluations rely on region-specific or single-dimensional indicators, often neglecting the systemic interactions among cities within agglomerations.^27^^,^^28^ Specifically, a recent study has constructed multi-dimensional indicators for evaluating the sustainable development of urban agglomerations and applied the geographical detector to assess the spatial heterogeneity of sustainability. However, this study did not consider urban agglomerations as distinct geographical units with interdependent relationships.^27^ In contrast, our study proposes a multi-level coupling coordination model that integrates various influence mechanisms among urban agglomerations, reflecting their nature as interdependent systems.

Third, the literature on sustainable development assessments remains fragmented, lacking a cohesive structure for indicator validation and integration.^29^ To address this, we propose a comprehensive “subsystem–element–indicator–SDGs” network framework, combined with redundancy and sensitivity analyses to ensure robustness and adaptability. This methodological contribution not only enhances internal consistency but also establishes meaningful connections with international SDG standards, responding to the need for comparability and policy relevance in diverse socio-economic contexts.

In summary, the “indicator-assessment-verification” framework for sustainable development of urban agglomerations proposed in this paper provides a clear and practical approach to systematically understanding and evaluating sustainable development patterns at the regional scale. This framework can directly inform both subsequent academic research and real-world policy decisions. Given the diversity in social, cultural, and economic contexts across different regions and countries, the interpretation and practical application of sustainable development indicators may vary significantly. The “subsystem-element-indicator” structure introduced in this study offers considerable flexibility and adaptability, allowing it to be tailored and adjusted according to the specific conditions and needs of different urban agglomerations.

In practical policymaking scenarios, decision-makers can leverage this framework to develop indicator systems that align closely with local characteristics. Through this process, they can effectively identify relationships between indicators, assess indicator redundancy and sensitivity, and pinpoint strengths and weaknesses within regional development. This nuanced understanding facilitates the formulation of targeted and evidence-based policy measures, addressing key development challenges and optimizing resource allocation and spatial planning. Consequently, this research not only lays a methodological foundation for future studies but also enhances the practical applicability of the findings by providing explicit guidance for real-world sustainable development strategies.

### Limitations of the study

This study has several limitations. This paper defines the study area according to the boundaries of urban agglomerations delineated in China’s 14th Five-Year Plan. However, urban agglomerations often emerge spontaneously through complex socio-economic and spatial interactions, which may not align with administrative or planning boundaries. Furthermore, due to the relatively limited scope of our study, we employed manual verification to correct outliers and ensure data quality. Future research could explore automated anomaly detection techniques, such as machine learning-based algorithms, which can identify anomalies dynamically without the need for predefined thresholds. Additionally, to further assess the stability and adaptability of the indicator system, future studies could incorporate multi-scenario simulations. By simulating different data conditions, such as variations in data quality, regional disparities, and extreme cases of missing or inconsistent data, the framework’s ability to maintain robustness under varying conditions can be systematically evaluated.

Although we have demonstrated the applicability and effectiveness of our indicator system and method in the middle reaches of the Yangtze River urban agglomeration region, we acknowledge that this case study alone does not capture the substantial regional differences that exist across China’s diverse urban agglomerations. In future research, we plan to expand the scope of analysis to include multiple urban agglomerations across different regions and development stages. Such comparative studies will help further test the robustness of our methodological framework and enhance its applicability at national and global scales. Furthermore, this study primarily employs an empirically based descriptive approach to construct the relationship between indicators and SDGs, which may lead to some complex relationships not being fully quantified. Future research could investigate sustainable development on a broader scale, either at the national or global level, or conduct more in-depth studies that examine both surface and subsurface dynamics.^30^^,^^31^

## Resource availability

### Lead contact

Requests and questions should be directed to Lizhe Wang (lizhe.wang@gmail.com).

### Materials availability

No materials were used directly as part of this study.

### Data and code availability

• The data used in this study are all available from public resources that have been appropriately cited within the manuscript and supplemental information.
• All custom code can be available on request from the lead contact.
• Any additional information required to reanalyze the data reported in this paper is available from the lead contact upon request.

## Acknowledgments

This work was supported by the National Natural Science Foundation of China (U21A2013).

## Author contributions

Y.S.: Conceptualization, methodology, investigation, writing – original draft, writing – review and editing; W.H.: Writing – review and editing; M.J.: Writing – review and editing; L.W.: Conceptualization, methodology, investigation, supervision.

## Declaration of interests

The authors declare no competing interests.

## STAR★Methods

### Key resources table

**Table:** 

REAGENT or RESOURCE	SOURCE	IDENTIFIER
**Deposited data**		

Urban agglomeration sustainability assessment data	This paper	N/A

**Software and algorithms**		

Python	Version 3.8.10	https://www.python.org/downloads/release/python-3810
R	Version 4.4.1	https://cran.rstudio.com/bin/windows/base/
ArcGIS Pro	Version 3.2	https://pro.arcgis.com/en/pro-app/latest/get-started/download-arcgis-pro.htm
Geoda	Version 1.22	https://geodacenter.github.io/index-cn.html

### Method details

#### Study area and data sources

China’s five major urban agglomerations are the Beijing-Tianjin-Hebei urban agglomeration, the Yangtze River Delta urban agglomeration, the Pearl River Delta urban agglomeration, the Chengdu-Chongqing urban agglomeration, and the city agglomeration in the middle reaches of the Yangtze River (Figure 1B). These regions concentrate many industries and populations and are the key engines of China’s socio-economic development. The Beijing-Tianjin-Hebei urban agglomeration has the largest difference in per capita GDP between cities and towns, the Yangtze River Delta urban agglomeration ranks first among China’s major urban agglomerations in terms of total GDP, the Pearl River Delta urban agglomeration is the highest urban density urban agglomeration in China, the Chengdu-Chongqing urban agglomeration is the largest urban agglomeration in the western region in terms of total GDP. The Yangtze River Middle Urban Agglomeration (YRMUA), comprising 31 cities (Figure 1A), is the largest national urban agglomeration in terms of spatial extent and is distinguished by pronounced conflicts and synergies among diverse development goals. Centered around the cross-provincial core cities of Wuhan, Changsha, and Nanchang, it also encompasses numerous smaller surrounding cities. This region plays a critical role in promoting economic development in central China, covering an area of 326,100 km^2^. Accordingly, this study selects the YRMUA as a representative case to examine and elucidate the complex interrelationships within urban agglomerations.

The comprehensive pattern recognition and SDG network analysis dataset for China’s SDGs and includes individual SDG performance at the city level.^32^ This dataset utilizes big data to track the overall SDG progress of 337 Chinese cities. For instance, the calculation of SDG 9 is based on nine specific indicators, including per capita passenger transport volume and the number of patents granted. The dataset of urban agglomeration sustainable development assessment indicators proposed in this paper consists of two major types. Statistical data provide the majority of social and economic indicators at the city level, which are sourced from the Chinese Urban Statistical Yearbook, the China Regional Economic Statistical Yearbook, and the Statistical Yearbook of Hubei, Hunan, and Jiangxi provinces for 2020. The second type consists of remote sensing data products, which complement statistical data by providing surface information, such as precipitation and topography. Additionally, the population mobility dataset is employed to represent material flow patterns and is obtained from the Baidu Migration platform.

#### Data preprocessing

Missing values in statistical data are especially common in smaller cities, such as Xiantao, Qianjiang, and Tianmen. To address this issue, this study proposes a spatial distance-weighted imputation strategy. This strategy identifies the five cities nearest to the target city by calculating spatial distances and assigning weights to these cities based on their proximity, thus facilitating the imputation of missing values. For the statistical analysis of remote sensing data products at the urban scale, detailed calculation methods are outlined in Table S1. For instance, the urban night light level indicator was calculated by integrating pixel-level brightness values over the urban area, reflecting the total luminous output of a city at night.

#### SDGs spatial pattern understanding and network constructing

To analyze the spatial patterns of the SDG scores, this study utilizes the sustainable development scores of 253 Chinese cities.^32^ The Kriging interpolation method in ArcGIS is employed to convert point data into raster format. This study utilizes the Pearson correlation coefficients among 16 SDGs across 253 cities in China to construct the correlation matrix of the SDG network. The SDG network is estimated using the Gaussian graphical model with graphical lasso based on the extended BIC criterion algorithm.^33^ In this network, the nodes represent the 16 interactive SDGs (excluding SDG 14), while the links between the nodes indicate the positive or negative correlations between them, along with their weights (Figure 2A). SDG 14, which focuses on life below water, primarily relies on extensive policy coordination at the global or regional level. In contrast, the sustainable development of urban agglomerations emphasizes local and regional ecosystems, economic dynamics, and social factors. Therefore, due to the differences in spatial scale between the two, this study does not provide an in-depth exploration of SDG 14.

Additionally, this study assesses the importance of nodes within the SDG network through various centrality measures, including Degree Centrality, Closeness Centrality, Betweenness Centrality, and Expected Influence. At the urban agglomeration scale, the edges of the SDG network are defined by Pearson correlation coefficients, while the modular community structure is identified using the Cluster Fast Greed algorithm. For the population mobility network, edges are defined by the migration scale in 2020, and the Modularity algorithm in Gephi is employed to detect communities.^34^ Local Moran’s I is utilized to assess spatial autocorrelation between each location and its surrounding neighborhood, thereby identifying the spatial patterns of sustainable development within urban agglomerations.

#### Development and selection of sustainable development indicators

The proposed “Subsystem-Element-Indicator” framework developed based on the Triple Bottom Line theory, the DPSIR model, and the PSR model, aims to decompose the complex system of urban agglomerations. Since urban agglomerations serve as hubs for human activities and Sustainable Development Goal (SDG) 11 emphasizes “inclusiveness, security, and resilience,” we have modified the traditional “social-economic-environmental” framework. Specifically, we separated the “natural environment” and “human settlement environment” into distinct subsystems. Furthermore, the “social” and “economic” dimensions were integrated into a unified “socioeconomic” dimension. Consequently, the framework consists of three subsystems: “socioeconomic,” “natural environment,” and “human settlement environment.” Based on data availability, key elements for each subsystem are identified, followed by the development of specific, quantifiable indicators. (as detailed in Table S1).

(1) The socioeconomic subsystem, which encompasses the social and economic development goals of urban agglomerations. The SES comprises both economic and social elements. Economic elements include economic growth, economic structure, employment, income levels, and the urban-rural income gap. The social elements encompass urbanization, education, healthcare security, social security, and technological innovation. The proposed indicators per capita were calculated based on the regional resident population, encompassing a total of 11 indicators.
(2) The human settlement subsystem, which addresses the living environment of urban agglomeration residents and related infrastructure development goals, focusing on residential conditions, infrastructure, and transportation within a city.^35^
(3) The natural environment subsystem, which concentrates on the natural resources and environmental conservation goals of urban agglomerations. The NES comprises geographical, ecological, and resource elements, including ten indicators: terrain, plant cover, temperature, precipitation, pollutants, forests, and arable land.

#### Comprehensive assessment methodologies

An important concept in information theory is entropy, which measures the degree of disorder or randomness and reflects the level of uncertainty within a system.^36^ This concept is utilized to quantify the contribution of individual indicators during the weighting process. In the context of this study, n refers to cities and m denotes indicators.

The proportion $P_{ij}$ of indicator $X_{j}$ from city i is calculated as follows:

(Equation 1) $$\begin{array}{c} P_{ij}=\frac{X_{ij}}{\sum_{i=1}^{n}X_{ij}} \end{array}$$

The information entropy $E_{j}$ for indicator $X_{j}$ is calculated as follows:

(Equation 2) $$\begin{array}{c} E_{j}=-\frac{{\ln\left(n\right)}^{-1}}{\sum_{i=1}^{n}p_{ij}\;\ln\left(p_{ij}\right)} \end{array}$$

The weight $W_{j}$ for indicator $X_{j}$ is calculated as follows:

(Equation 3) $$\begin{array}{c} W_{j}=\frac{1-E_{j}}{\sum_{i=1}^{m}\left(1-E_{j}\right)} \end{array}$$

The sustainable development index ${SDI}_{s}$ of subsystem s is calculated as follows:

(Equation 4) $$\begin{array}{c} {SDI}_{s}=\sum_{j=1}^{m}W_{j}\times X_{ij} \end{array}$$

The calculations in Equations 1, 2, 3, and 4 are performed at the three subsystem levels of the city, resulting in subsystems SDI for the $SES_{i}$, $NES_{i}$, and $HSS_{i}$ for city i. To obtain USDI, the indicators $W_{SES}$, $W_{NES}$ and $W_{HSS}$ represent the weights attributed to the three subsystems. Each weight is assigned a value of $\frac{1}{3}$. The USDI is defined as:

(Equation 5) $$\begin{array}{c} USDI_{i}=\sqrt{\left(W_{SES}\cdot SES_{i}+W_{NES}\cdot NES_{i}+W_{HHS}\cdot HSS_{i}\right)} \end{array}$$

The multi-level coupling and coordination degree quantification method explains the interrelationships between subsystems, which can be seen in both urban systems and urban agglomeration systems.^37^^,^^38^ To evaluate the coupling and coordination degree between cities more accurately, inverse distance weighting (IDW) was used to determine the weights.^39^ The measure of intra-city coupling degree is defined as:

(Equation 6) $$\begin{array}{c} {IC}_{i}=\frac{1}{3}\left(\sqrt{\frac{NES_{i}\cdot SES_{i}}{{\left(\frac{NES_{i}+SES_{i}}{2}\right)}^{2}}}+\sqrt{\frac{NES_{i}\cdot HSS_{i}}{{\left(\frac{NES_{i}+HSS_{i}}{2}\right)}^{2}}}+\sqrt{\frac{SES_{i}\cdot HSS_{i}}{{\left(\frac{SES_{i}+HSS_{i}}{2}\right)}^{2}}}\right) \end{array}$$

The definition of the degree of inter-city coupling degree measure is defined as follows:

(Equation 7) $$\begin{array}{c} {EC}_{ik}=\sqrt{\frac{{USDI}_{i}\cdot {USDI}_{k}}{{\left(\frac{{USDI}_{i}+{USDI}_{k}}{2}\right)}^{2}}} \end{array}$$

The definition to determine both intra-city and inter-city coupling degree based on the IDW method is as follows:

(Equation 8) $$\begin{array}{c} IEC_{i}=\mu \cdot IC_{i}+\lambda \cdot \sum_{k=1}^{n}w_{ik}\cdot EC_{ik},i\neq k \end{array}$$

where n is the total number of cities, and k is an integer ranging from 1 to n, where i≠k. We select the 10 cities closest to city i by distance from among the n cities. The contribution coefficients for intra-city and inter-city coupling degree are μ and λ, respectively, with both being 0.5:

(Equation 9) $$\begin{array}{c} w_{ik}=\frac{d_{ik}^{-p}}{\sum_{k=1}^{n}d_{ik}^{-p}} \end{array}$$

The definition of $W_{ik}$ based on IDW is as above, where $d_{ik}$ is the distance between the administrative centers of city i and city k, and p is the exponent of this distance, usually taken as 2. The definition of intra-city coordination degree is as follows:

(Equation 10) $$\begin{array}{c} IT_{i}=\alpha \cdot NES_{i}+\beta \cdot SES_{i}+\gamma \cdot HSS_{i} \end{array}$$

where α, β, and γ are the weight coefficients for the natural environment, socio-economic and human settlement subsystems, respectively, while the weight coefficient is $\frac{1}{3}$.

The definition of the degree of inter-city coordination is as follows:

(Equation 11) $$\begin{array}{c} ET_{ik}=\frac{{USDI}_{i}+{USDI}_{k}}{2} \end{array}$$

considering both the degree of coordination within and between cities:

(Equation 12) $$\begin{array}{c} IET_{i}=\mu \cdot IT_{i}+\lambda \cdot \sum_{k=1}^{n}w_{ik}\cdot ET_{ik},i\neq k \end{array}$$

The definition of the multi-level coupling coordination degree $D_{i}$ is as follows:

(Equation 13) $$\begin{array}{c} D_{i}=\sqrt{IEC_{i}\cdot IET_{i}} \end{array}$$

where ${IEC}_{i}$ is the intra-city and inter-city coupling degree, and ${IET}_{i}$ is the intra-city and inter-city coordination degree. The formula for sustainable development evaluation for urban agglomerations is as follows:

(Equation 14) $$\begin{array}{c} UASI=\sqrt{\alpha \cdot USDI+\beta \cdot D} \end{array}$$

Here α and β represent the weights of USDI and D, respectively, both of which are 0.5.

#### Validation techniques and robustness check

Indicators were aligned with the SDGs to show the SDG priorities that the indicators in this paper focus on. One indicator is aligned with one direct SDG and two indirect SDGs in Table S2. This ensures that indicator systems built for specific issues are consistent with global frameworks and standards. Before validating the indicators and methodologies, the maximin principle was used to standardize the data.

This study uses the average correlation coefficient of the correlation coefficient matrix to evaluate the redundancy (RD) of the indicator,^25^ while the Monte Carlo approach is used to assess the sensitivity of the indicators^40^:

(Equation 15) $$\begin{array}{c} Cov\left(X_{i},X_{j}\right)=E\left[\left(X_{i}-\mu_{i}\right)\left(X_{j}-\mu_{j}\right)\right] \end{array}$$

Covariance is a statistical measure used to evaluate the linear relationship between two variables. Here, $\mu_{i}$ and $\mu_{j}$ represent the meaning of the i and j indicators. The variance of indicator $X_{i}$ is represented by $Var\left(X_{i}\right)$, measuring the level of dispersion of the indicators within the study area:

(Equation 16) $$\begin{array}{c} Var\left(X_{i}\right)=\frac{1}{n-1}\sum_{j=1}^{n}{\left(X_{ij}-\bar{X_{i}}\right)}^{2} \end{array}$$

The RD of the indicators is calculated based on the average correlation coefficient:

(Equation 17) $$\begin{array}{c} RD=\frac{1}{\frac{n\left(n-1\right)}{2}}\sum_{i=1}^{n}\;\sum_{j=i+1}^{n}\;\frac{\text{Cov}\left(X_{i},X_{j}\right)}{\sqrt{\text{Var}\left(X_{i}\right)\cdot \text{Var}\left(X_{j}\right)}} \end{array}$$

The RD measures the overall redundancy level of a system. To evaluate the contribution of individual indicators to redundancy, we employ a stepwise elimination method. Specifically, each indicator $X_{i}$ is removed one at a time, and the redundancy degree is recalculated as $RD_{-i}$. If the $RD_{-i}$ decreases significantly after removing a certain indicator, it indicates that this indicator contributes substantially to the overall redundancy. The individual redundancy contribution $\Delta RD_{i}$ is defined as:

(Equation 18) $$\begin{array}{c} \Delta RD_{i}=RD-RD_{-i} \end{array}$$

By simulating the uncertainty or randomness factors in the problem multiple times, this study explored the corresponding changes in the evaluation results when one or more indicators varied. To introduce controlled perturbations, we implemented a random selection perturbation mechanism, where a fixed adjustment of 0.1 was applied to the original data based on a stochastic selection process. The sensitivity of the indicators is defined as follows:

(Equation 19) $$\begin{array}{c} Sen=\frac{1}{N}\sum_{i=1}^{N}\left(E^{\prime }-E\right) \end{array}$$

Here, N represents the times of simulate experience, set to 1000. The $E^{\prime }$ denotes the evaluation score after perturbation and E is the original evaluation score.

To integrate the redundancy contribution and sensitivity of indicators, we first normalize both to ensure they are on the same scale for comparability. Based on this, we construct an Impact Index to quantify the overall influence of an indicator within the validation framework, which is defined as:

(Equation 20) $$\begin{array}{c} I_{i}=w_{1}\times \Delta RD_{i}+w_{2}\times Sen_{i} \end{array}$$

Here, $w_{1}$ and $w_{2}$ are weight coefficients, with a default setting of 0.5, assigning equal importance to redundancy contribution and sensitivity.

To reveal the relationship between the indicators and the SDGs, the validation methodology consists of the following three steps.

(1) **Establish a network of associations between the indicators and the SDGs.** This network comprises five key levels: system layer, subsystem layer, objective target layer, virtual layer and operation indicator layer. The operation indicator layer encompasses multiple specific, well-defined and practically measurable indicators, denoted as $D_{k}$. The virtual layer contains abstract elements $E_{j}$, which logically extend the operable indicators $D_{k}$ and offer a multi-level interpretive relationship,^41^ where $E_{j}$ comprises one or more $D_{k}$. The objective target layer represents our assessment goals, specifically the SDGs. The subsystem layer contains different key areas, represented by $S_{i}$.
(2) **Define the strength between the indicators and the SDGs.** Due to variations in data availability across different countries or regions, where not all indicators are directly attainable, this study employed elements from urban agglomeration sustainable indicators as a virtual layer.The linkage weight of a specific indicator $D_{k}$ is represented by $W_{ik}$ in the indicators and SDGs, signifying causal relationships or ecological processes between indicators.^42^ This weight is categorized into three types: 0 (no connection), 0.5 (indirect connection) and 1 (direct connection). Through statistical analysis of the connections, $W_{ik}$ between indicator $D_{k}$ and SDGs within each element $E_{j}$ of the virtual layer, the degree of linkage $W_{ij}^{\prime }$ between element $E_{j}$ and SDGs is determined:

(Equation 21) Wij'=∑kWik·1{Dk⊆Ej}

(3) **Measuring the focus of indicators on SDGs.** Given the complexity of the SDGs, indicator $D_{k}$ contributes to the realization of multiple SDGs. By measuring the degree of connection $W_{ij}^{\prime }$ between element $E_{j}$ and the SDGs, it is feasible to obtain the degree of emphasis of the SDGs in the indicators. For instance, the focus on SDG 1 is defined as $G_{1}$:

(Equation 22) $$\begin{array}{c} G_{1}=\frac{\sum W_{1j}^{\prime }}{\sum W_{1j}^{\prime }+\sum W_{2j}^{\prime }+\cdots +\sum W_{17j}^{\prime }} \end{array}$$

#### Indicator normalization

Big data on the Earth provide a wide and diverse source for SDG monitoring, contributing to the more comprehensive and accurate assessment and advancement of the SDGs.^43^^,^^44^ This study used statistical data and remote sensing image data of 31 cities in the middle reaches of the Yangtze River in 2020. The proposed indicators include both positive and negative indicators. Positive indicators include D1–5, D7–11, D13–14, D19–27, D29-30, D33, D29–30, D35 and D37, while negative indicators include D6, D12, D15–18, D28, D31–32, D34, D36 and D38. Each indicator was standardized as minimum-maximum to ensure that the data ranged from 0 to 1, which is conducive to comprehensive analysis of indicators in different units or dimensions. The normalization formula is as follows:

Positive indicator:

(Equation 23) $$\begin{array}{c} x=\frac{x_{i}-\min\;x_{i}}{\max\;x_{i}-\min\;x_{i}} \end{array}$$

Negative indicator:

(Equation 24) $$\begin{array}{c} x=1-\frac{x_{i}-\min\;x_{i}}{\max\;x_{i}-\min\;x_{i}} \end{array}$$

### Quantification and statistical analysis

In this study, we used R, ArcGIS Pro, Python and Geoda for data analysis.

ArcGIS Pro (3.2) was used for sampling and coordinate transformation of all remote sensing data to standardize the data resolution and coordinate system. The “Zonal Statistics as Table” tool was applied to calculate the parameter values at the prefecture-level city scale.

Python (3.8.10) was used to process missing data and to implement the methodological framework for sustainability assessment, validation, and robustness checks.

R (4.4.1) was used to estimate the national SDG network using the bootnet package, applying Spearman and EBICglasso algorithm. The modular community structure of the SDG network at the urban agglomeration level was detected using the Cluster Fast Greedy algorithm implemented in the igraph package.

GeoDa (1.22) was used to describe the sustainable development pattern of the urban agglomeration in the middle reaches of the Yangtze River described by Moran I.

All statistical details, including sample sizes, correlation values, and clustering results, are provided in the figure legends, Results section, and Supplementary Tables.
