# Supplementary material for: Indicators and methodologies for assessing urban agglomeration sustainability
Source: iScience. 2025 Jun 18;28(7):112927. doi: 10.1016/j.isci.2025.112927 (PMC12269625; doi:10.1016/j.isci.2025.112927)
Supplement: Document S1. Figure S1 and Tables S1–S5 [file mmc1.pdf]

iScience, Volume *28*

Supplemental information

Indicators and methodologies for assessing urban agglomeration sustainability

Yinghui Shao, Wei Han, Min Jin, and Lizhe Wang

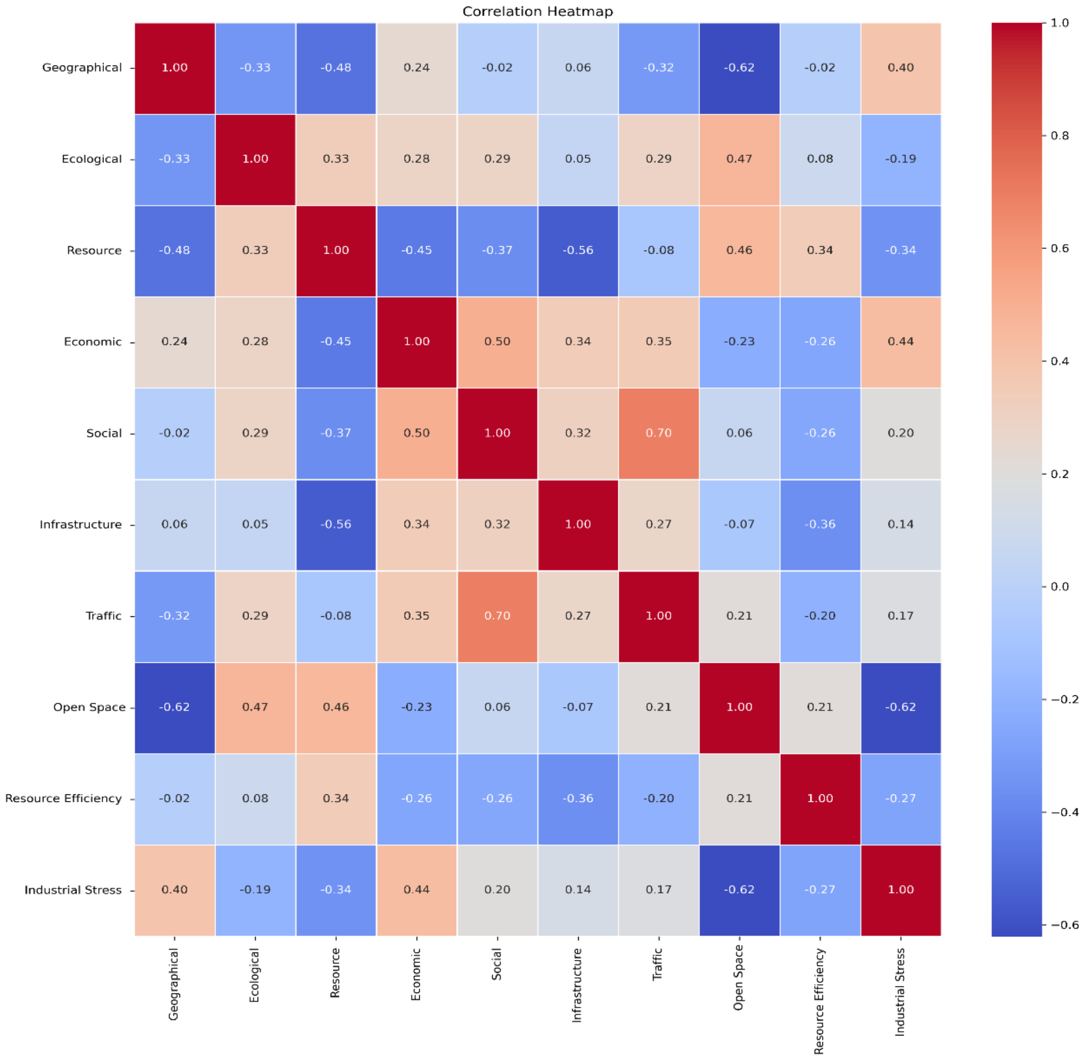

Figure S1 Correlation analysis at element scale across subsystem, Related to Validation of Sustainable Development Indicators and Methodologies.

Table S1. Indicators for the sustainable development of urban agglomerations, Related to STAR METHODS.

Subsystem	Element	ID	Indicator	Computational formula
Socio-economic subsystem	Economic element	D1	Annual growth rate of GDP per capita	——
		D2	The proportion of tertiary industry in GDP	——
		D3	Urban registered unemployment rate	——
		D4	Per capita disposable income of urban residents	——
		D5	Per capita disposable income of rural residents	——
		D6	Urban-rural income gap	D 6 = D 4/D 5*100
		D7	Urban night light level	D 7 = Luminous night light * area
	Social element	D8	Education expenditure per capita	D 8 = Total education expenditure/resident population
		D9	Hospital beds per capita	D 9 = Total hospital beds/resident population
		D10	Participation rate of basic endowment insurance for urban employees	D 10 = Insured employees/resident population
		D11	Science and technology expenditure per capita	D 11 = Technology expenditure/resident population
Natural environment subsystem	Geographical element	D12	Slope	Arcgis 3D analyst tools
		D13	Distance to water resources	Arcgis spatial analyst tools
	Ecological element	D14	NDVI difference	——
		D15	Temperature change rate	(Average temperature_2020-average temperature_2019)/Average temperature_2019
		D16	Precipitation rate of change	(Mean annual precipitation_2020-mean annual precipitation_2019)/mean annual precipitation_2019
		D17-18	Concentration of ozone and sulfur dioxide	Arcgis spatial analyst tools
		D19	Mountain biodiversity	D 19 = Total number of species
	Resource element	D20	Forest cover rate	Arcgis spatial analyst tools
		D21	Water resources per capita	D 21 = Total water resources/resident population
		D22	Cultivated land proportion	Arcgis spatial analyst tools
Human settlement subsystem	Urban infrastructure element	D23	Gas penetration rate	——
		D24	Water supply penetration rate	——
		D25	Water supply pipe density	——
	Urban traffic element	D26	Road freight volume	——
		D27	Road area per capita	——
		D28	Public tram ridership	——
	Urban open space element	D29	PM2.5 concentration	D 29 = Average PM2.5 concentration
		D30	Green park area per capita	——
		D31	Urban green coverage rate	——
	Resource efficiency element	D32	Energy consumptions per GDP	——
		D33	CO2 emission	——
		D34	Comprehensive utilization rate of industrial solid waste	——
		D35	Sewage treatment plant treatment rate	——
	Industrial production stressors element	D36	Industrial smoke and dust emissions per industrial added value	D 36 = Industrial smoke and dust emissions/industrial added value
		D37	Industrial effluent discharge per industrial added value	D 37 = Industrial effluent discharge/industrial added value
		D38	Industrial sulfur dioxide emissions per industrial added value	D 38 = Industrial sulfur dioxide emissions /industrial added value

Table S2. Relevance of indicators for Sustainable Development Goals and data securement, Related to STAR METHODS.

ID	Indicator	Data sources	Direct Goal	Indirect Goal
D1	Annual growth rate of GDP per capita	Chinese Urban Statistical Yearbook	SDG 8	SDG 1, SDG 9
D2	The proportion of tertiary industry in GDP	Chinese Urban Statistical Yearbook	SDG 8	SDG 1, SDG 9
D3	Urban registered unemployment rate	China Regional Economic Statistical Yearbook	SDG 8	SDG 1, SDG 10
D4	Per capita disposable income of urban residents	Statistical Yearbook of Hubei, Hunan, and Jiangxi provinces	SDG 8	SDG 1, SDG 10
D5	Per capita disposable income of rural residents	Statistical Yearbook of Hubei, Hunan, and Jiangxi provinces	SDG 8	SDG 1, SDG 2
D6	Urban-rural income gap	Statistical Yearbook of Hubei, Hunan, and Jiangxi provinces	SDG 10	SDG 1, SDG 8
D7	Urban night light level	A Prolonged Artificial Nighttime-Light Dataset of China (1984–2020)	SDG 11	SDG7, SDG 13
D8	Education expenditure per capita	Statistical Yearbook of Hubei, Hunan, and Jiangxi provinces	SDG 4	SDG 1, SDG 5
D9	Hospital beds per capita	Statistical Yearbook of Hubei, Hunan, and Jiangxi provinces	SDG 3	SDG 8, SDG 10
D10	Participation rate of basic endowment insurance for urban employees	Statistical Yearbook of Hubei, Hunan, and Jiangxi provinces	SDG 1	SDG 8, SDG 10
D11	Science and technology expenditure per capita	Statistical Yearbook of Hubei, Hunan, and Jiangxi provinces	SDG 9	SDG 4, SDG 10
D12	Slope	AlosDEM (search.asf.alaska.edu)	SDG 15	SDG 6, SDG 13
D13	Distance to water resources	OpenStreetMap (openstreetmap.org)	SDG 6	SDG 3, SDG 11
D14	NDVI difference	MOD17A3HGF Version 6.0 (lpdaac.usgs.gov)	SDG 15	SDG 6, SDG 1
D15	Temperature change rate	NOAA (www.noaa.gov)	SDG 13	SDG 7, SDG 11
D16	Precipitation rate of change	China 1 km resolution monthly precipitation datasets	SDG 6	SDG 11, SDG 13
D17-18	Concentration of ozone or sulfur dioxide	China 1 km resolution high air pollutant (CHAP) datasets (weijing-rs.github.io/product)	SDG 13	SDG 3, SDG 11
D19	Mountain biodiversity	GBIF Datasets (www.gbif.org)	SDG 15	SDG 2, SDG 13
D20	Forest cover rate	China Land Cover Dataset	SDG 15	SDG 6, SDG 13
D21	Water resources per capita	Statistical Yearbook of Hubei, Hunan, and Jiangxi provinces	SDG 6	SDG 3, SDG 7
D22	Cultivated land proportion	China Land Cover Datasets	SDG 2	SDG 13, SDG 15
D23	Gas supply penetration rate	Chinese Urban Construction Statistical Yearbook	SDG 7	SDG 3, SDG 13
D24	Water supply penetration rate	Chinese Urban Construction Statistical Yearbook	SDG 6	SDG 3, SDG 7
D25	Water supply pipe density	Chinese Urban Construction Statistical Yearbook	SDG 6	SDG 3, SDG 7
D26	Road freight volume	Statistical Yearbook of Hubei, Hunan, and Jiangxi provinces	SDG 9	SDG 8, SDG 11
D27	Road area per capita	Statistical Yearbook of Hubei, Hunan, and Jiangxi provinces	SDG 9	SDG 8, SDG 11
D28	Public tram ridership	Chinese Urban Statistical Yearbook	SDG 11	SDG 9, SDG13
D29	PM2.5 concentration	Surface PM2.5 datasets(surface-pm2-5)	SDG 3	SDG 11, SDG 13
D30	Green park area per capita	Chinese Urban Construction Statistical Yearbook	SDG 11	SDG 3, SDG 15
D31	Urban green coverage rate	Chinese Urban Construction Statistical Yearbook	SDG 11	SDG 3, SDG 15
D32	Energy consumptions per GDP	Statistical Yearbook of Hubei, Hunan and Jiangxi provinces	SDG 7	SDG 8, SDG 13
D33	CO_2 emissions	CO_2 emissions from Chinese cities datasets	SDG 13	SDG 7, SDG 11
D34	Comprehensive utilization rate of industrial solid waste	Statistical Yearbook of Hubei, Hunan and Jiangxi provinces	SDG 12	SDG 9, SDG 13
D35	Sewage treatment plant treatment rate	Statistical Yearbook of Hubei, Hunan and Jiangxi provinces	SDG 6	SDG 3, SDG 11
D36	Industrial smoke and dust emissions per industrial added value	Statistical yearbook of Hubei, Hunan and Jiangxi provinces	SDG12	SDG9, SDG13
D37	Industrial effluent discharge per industrial added value	Statistical yearbook of Hubei, Hunan and Jiangxi provinces	SDG6	SDG9, SDG13
D38	Industrial sulfur dioxide emissions per industrial added value	Statistical yearbook of Hubei, Hunan and Jiangxi provinces	SDG13	SDG9, SDG11

Table S3. Results of sustainable development assessment, Related to Figure 5.

City	NES	SES	HSS	U	IC	EC	IEC	IT	ET	IET	D	USAI
Nanchang	0.65	0.48	0.50	0.74	0.99	0.70	0.85	0.54	0.49	0.515	0.82	0.883
Jingdezhen	0.56	0.32	0.54	0.69	0.98	0.77	0.87	0.47	0.51	0.494	0.83	0.870
Pingxiang	0.49	0.38	0.46	0.67	1.00	0.83	0.91	0.44	0.56	0.503	0.84	0.868
Jiujiang	0.58	0.19	0.54	0.66	0.91	0.71	0.81	0.44	0.46	0.448	0.79	0.853
Xinyu	0.41	0.29	0.50	0.63	0.98	0.81	0.89	0.40	0.53	0.464	0.82	0.853
Yingtan	0.49	0.27	0.56	0.66	0.97	0.83	0.90	0.44	0.56	0.498	0.84	0.866
Ji'an	0.66	0.15	0.47	0.65	0.87	0.77	0.82	0.42	0.51	0.468	0.80	0.853
Yichun	0.54	0.23	0.63	0.68	0.94	0.83	0.88	0.47	0.56	0.512	0.83	0.871
Fuzhou	0.53	0.25	0.67	0.69	0.94	0.79	0.86	0.48	0.54	0.511	0.83	0.873
Shangrao	0.63	0.17	0.45	0.65	0.90	0.80	0.85	0.42	0.53	0.473	0.81	0.855
Wuhan	0.36	0.80	0.58	0.76	0.96	0.87	0.91	0.58	0.59	0.582	0.87	0.902
Huangshi	0.24	0.15	0.49	0.54	0.92	0.93	0.92	0.29	0.52	0.403	0.81	0.822
Yichang	0.46	0.34	0.43	0.64	0.99	0.77	0.88	0.41	0.48	0.446	0.81	0.854
Xiangyang	0.27	0.24	0.48	0.58	0.97	0.74	0.86	0.33	0.44	0.386	0.79	0.826
Ezhou	0.25	0.14	0.45	0.53	0.92	0.99	0.96	0.28	0.55	0.416	0.83	0.824
Jingmen	0.39	0.27	0.37	0.58	0.99	0.82	0.91	0.34	0.49	0.417	0.81	0.836
Xiaogan	0.42	0.13	0.38	0.56	0.90	0.85	0.87	0.31	0.51	0.410	0.80	0.824
Jingzhou	0.49	0.17	0.34	0.58	0.94	0.81	0.87	0.34	0.48	0.408	0.80	0.830
Huanggang	0.45	0.05	0.51	0.58	0.71	0.99	0.85	0.34	0.55	0.444	0.80	0.832
Xianning	0.44	0.02	0.56	0.59	0.60	0.76	0.68	0.34	0.46	0.399	0.74	0.813
Xiantao	0.54	0.22	0.46	0.64	0.95	0.85	0.90	0.41	0.53	0.469	0.83	0.856
Qianjiang	0.52	0.17	0.43	0.61	0.92	0.87	0.89	0.37	0.53	0.450	0.82	0.846
Tianmen	0.50	0.18	0.30	0.57	0.94	0.88	0.91	0.32	0.52	0.424	0.82	0.832
Changsha	0.57	0.89	0.65	0.84	0.99	0.87	0.93	0.70	0.67	0.684	0.90	0.931
Zhuzhou	0.55	0.47	0.65	0.74	0.99	0.95	0.97	0.55	0.68	0.618	0.89	0.904
Xiangtan	0.58	0.40	0.38	0.67	0.99	0.95	0.97	0.45	0.68	0.565	0.88	0.880
Hengyang	0.50	0.38	0.43	0.66	1.00	0.79	0.89	0.44	0.54	0.488	0.83	0.864
Yueyang	0.58	0.31	0.45	0.67	0.98	0.62	0.80	0.45	0.41	0.428	0.78	0.852
Changde	0.57	0.25	0.61	0.69	0.94	0.71	0.82	0.48	0.48	0.481	0.81	0.866
Yiyang	0.63	0.22	0.51	0.67	0.93	0.79	0.86	0.45	0.55	0.501	0.82	0.865
Loudi	0.65	0.17	0.43	0.64	0.89	0.81	0.85	0.42	0.55	0.482	0.82	0.855

Table S4. Results of the sensitivity test at the element level and total indicator level, Related to Validation of Sustainable Development Indicators and Methodologies.

**Indicator**	**Element**	**System**
Annual growth rate of GDP per capita	0.13	0.10
The proportion of tertiary industry in GDP	0.07	0.06
Urban registered unemployment rate	0.12	0.10
Per capita disposable income of urban residents	0.08	0.06
Per capita disposable income of rural residents	0.06	0.05
Urban-rural income gap	0.14	0.11
Urban night light level	0.08	0.05
Education expenditure per capita	0.13	0.09
Hospital beds per capita	0.13	0.09
Participation rate of basic endowment insurance for urban employees	0.13	0.08
Science and technology expenditure per capita	0.07	0.05
Slope	**0.45**	0.12
Distance to Water Resources	**0.36**	0.10
NDVI difference	0.16	0.12
Temperature change rate	0.18	0.14
Precipitation rate of change	0.15	0.11
Concentration of ozone	0.14	0.11
Concentration of sulfur dioxide	0.16	0.13
Mountain biodiversity	0.04	0.03
Forest cover rate	0.03	0.01
Water resources per capita	0.12	0.07
Cultivated land proportion	0.15	0.08
Gas supply penetration rate	**0.45**	**0.17**
Water supply penetration rate	**0.44**	**0.18**
Water supply pipe density	0.3	0.12
Road freight volume	0.15	0.06
Road area per capita	0.22	0.10
Public tram ridership	0.08	0.03
PM2.5 concentration	0.27	0.10
Green Park area per capita	0.22	0.09
Urban green coverage rate	0.21	0.08
Energy consumption per GDP	0.31	0.16
CO2 emissions	0.28	0.16
Comprehensive utilization rate of industrial solid waste	0.26	0.14
Sewage treatment plant treatment rate	0.31	0.16
Industrial smoke and dust emissions per industrial added value	0.39	0.15
Industrial effluent discharge per industrial added value	0.27	0.11
Industrial sulfur dioxide emissions per industrial added value	0.36	0.14

Table S5. Analysis of the indicator impact index base RC and Sen, Related to Validation of Sustainable Development Indicators and Methodologies.

ID	Subsystem	RC	Sen	Impact Index	Classification
1	SES	-0.00661	0.019826	0.716571	Low RC - Low Sen
2	SES	0.001555	0.011708	0.508209	High RC - Low Sen
3	SES	-0.00639	0.01971	0.712426	Low RC - Low Sen
4	SES	0.002784	0.012243	0.501955	High RC - Low Sen
5	SES	0.007079	0.009914	0.420066	High RC - Low Sen
6	SES	-0.00751	0.022621	0.766726	Low RC - High Sen
7	SES	0.004536	0.010521	0.457523	High RC - Low Sen
8	SES	-0.01067	0.018922	0.749583	Low RC - Low Sen
9	SES	-0.00758	0.017028	0.687603	Low RC - Low Sen
10	SES	-0.00451	0.016033	0.638611	Low RC - Low Sen
11	SES	0.00666	0.009731	0.422206	High RC - Low Sen
12	NES	-0.00904	0.023067	0.790415	Low RC - High Sen
13	NES	-0.00744	0.020679	0.738097	Low RC - High Sen
14	NES	-0.00536	0.024897	0.774817	Low RC - High Sen
15	NES	0.003486	0.027683	0.714529	High RC - High Sen
16	NES	-0.00866	0.022508	0.778101	Low RC - High Sen
17	NES	-0.00788	0.021766	0.758683	Low RC - High Sen
18	NES	-0.0066	0.025925	0.803587	Low RC - High Sen
19	NES	0.007066	0.006731	0.374748	High RC - Low Sen
20	NES	0.033468	0.001414	0	High RC - Low Sen
21	NES	0.000929	0.013915	0.54682	High RC - Low Sen
22	NES	-0.01071	0.016092	0.709642	Low RC - Low Sen
23	HSS	0.016706	0.034482	0.662023	High RC - High Sen
24	HSS	0.021312	0.03642	0.637581	High RC - High Sen
25	HSS	-0.00541	0.023518	0.755712	Low RC - High Sen
26	HSS	0.000946	0.011182	0.507598	High RC - Low Sen
27	HSS	-0.00799	0.019977	0.734408	Low RC - Low Sen
28	HSS	0.016789	0.005904	0.252896	High RC - Low Sen
29	HSS	-0.00774	0.02055	0.739718	Low RC - High Sen
30	HSS	-0.00634	0.018942	0.700915	Low RC - Low Sen
31	HSS	-0.00465	0.01603	0.640123	Low RC - Low Sen
32	HSS	0.005799	0.032367	0.75525	High RC - High Sen
33	HSS	0.005925	0.032934	0.761942	High RC - High Sen
34	HSS	-0.0043	0.027644	0.802039	Low RC - High Sen
35	HSS	0.006272	0.032694	0.754567	High RC - High Sen
36	HSS	0.000509	0.029919	0.780167	High RC - High Sen
37	HSS	-0.00551	0.021969	0.734744	Low RC - High Sen
38	HSS	0.001015	0.027207	0.735694	High RC - High Sen
